# Development of a prognostic risk model for clear cell renal cell carcinoma by systematic evaluation of DNA methylation markers: an update after ISUP/WHO 2022 classification

**DOI:** 10.1002/2056-4538.70030

**Published:** 2025-08-18

**Authors:** Selena Odeh, Iryna Samarska, Andres Matoso, Marcella MLL Baldewijns, Christina A Hulsbergen‐van de Kaa, Axel Zur Hausen, Manon van Engeland, Leo J Schouten, Kim M Smits

**Affiliations:** ^1^ Department of Pathology Research Institute for Oncology and Reproduction (GROW), Maastricht University Medical Center Maastricht The Netherlands; ^2^ Department of Pathology Johns Hopkins University Baltimore MD USA; ^3^ Department of Urology Johns Hopkins University Baltimore MD USA; ^4^ Department of Oncology Johns Hopkins University Baltimore MD USA; ^5^ Department of Histopathology University of Leuven Leuven Belgium; ^6^ Laboratory of Pathology East Netherlands (LABPON) Hengelo The Netherlands; ^7^ Department of Urology Medisch Spectrum Twente (MST) Enschede The Netherlands; ^8^ Department of Epidemiology Research Institute for Oncology and Reproduction (GROW), Maastricht University Medical Center Maastricht The Netherlands

**Keywords:** clear cell renal cancer, prognostic model, ISUP/WHO 2022 classification, survival

## Abstract

Although several prognostic models have been developed for clear cell renal cell carcinoma (ccRCC), these are still suboptimal and there is a need to identify additional prognostic biomarkers. Previously, we developed a prognostic model containing five DNA methylation markers (*NEFH*, *NEURL*, *GATA5*, *GREM1*, and *LAD1*) and clinicopathological characteristics based on the TNM 3rd edition and Fuhrman grading system. Here, we evaluated the effect of the recent ISUP/ 2022 WHO revisions on our previous prognostic model by incorporating the new ISUP/WHO standards, TNM 8th edition, and several novel prognostic factors (necrosis, lymphovascular invasion, sarcomatoid and rhabdoid features). Data from 308 ccRCC cases from the Netherlands Cohort Study were included for this study. Clinicopathological factors, novel prognostic factors, and the five methylation markers were analyzed for their individual and combined prognostic value using Kaplan–Meier analyses and Cox proportional hazard models. To compare models, the Akaike information criterion (AIC) and c‐statistic were used. All evaluated factors were statistically significantly associated with cause‐specific survival. The clinical model using the ISUP and TNM 8th edition performed similarly when compared to the Fuhrman/TNM 3rd edition model (AIC 592, c‐statistic 0.63 and AIC 595, c‐statistic 0.62, respectively). After addition of the five DNA methylation markers to the ISUP/TNM 8th model, this model was slightly improved (AIC 584, c‐statistic 0.70). The addition of necrosis and lymphovascular invasion (LVI) did not further improve these results (AIC 586, c‐statistic 0.71 and AIC 588, c‐statistic 0.71, respectively). Despite the individual prognostic significance of necrosis, LVI, the presence of sarcomatoid and/or rhabdoid differentiation, ISUP, and TNM 8th edition, these factors did not influence the performance of our prognostic model. The model including the five DNA methylation markers, age at diagnosis, sex, TNM stage (8th edition), ISUP grading, and tumor size was the best performing model, thereby highlighting the potential importance of molecular markers.

## Introduction

Renal cell carcinoma (RCC) is the most common malignant neoplasm of the kidney, accounting for 90% of kidney cancer cases [1]. It comprises several subtypes, among which clear cell RCC (ccRCC) is the most prevalent subtype (~75%), with papillary RCC (pRCC, ~15%) and chromophobe RCC (chRCC, ~5%) accounting for smaller proportions [1, 2, 3, 4, 5]. Patients with non‐metastatic ccRCC are treated with curative intent by (partial) nephrectomy, but 30% of these patients still develop metastases during follow‐up and ~10% die of disease progression within 5 years after surgery [6]. Currently, it is challenging to recognize these high‐risk patients upfront, especially on small biopsy samples, despite the availability of several clinical prognostic models such as the Tumor Node Metastasis (TNM) staging system [7], Fuhrman grading [8], the Stage, Size, Grade, Necrosis (SSIGN) Risk Score [9], and the University of California Los Angeles Integrated Staging System (UISS) [10]. Therefore, there is a need to identify and develop prognostic markers that can enhance the predictive performance of these prognostic models and that could have potential application on small biopsy material [11, 12]. Previously, we described a prognostic model comprising five DNA methylation markers (i.e., *GREM1*, *GATA5*, *LAD1*, *NEFH*, and *NEURL*) in combination with clinicopathological characteristics (i.e., age at diagnosis, sex, Fuhrman grade, tumor size, and TNM stage) [12]. This prognostic model performed slightly better in two tissue patient series compared to a model with solely clinicopathological characteristics (c‐statistics of 0.71 and 0.95 versus 0.65 and 0.86, respectively) [12].

In recent years, knowledge on the pathological, molecular, and clinical features of RCC has increased [13]. This was summarized in the new International Society of Urological Pathology (ISUP) 2013 Vancouver consensus [14] and subsequently published in the 4th and 5th editions of the World Health Organization (WHO) Classification of Tumors of the Urinary System and Male Genital Organs Bluebook in 2016 and 2022 [15, 16]. Additionally, the Vancouver consensus proposed a new ISUP grading system based on nucleolar size instead of Fuhrman grading as it is a more reproducible system [17, 18, 19] and the TNM staging has been revised currently in the 8th edition [7, 20, 21]. Moreover, several prognostic features such as the presence of sarcomatoid or rhabdoid differentiation [22], tumor necrosis (TN) [23], and lymphovascular (microvascular) invasion (LVI) [24] have been proposed.

Considering these advancements, we have previously re‐evaluated our population‐based series of RCC cases from the Netherlands Cohort Study on diet and cancer (NLCS) [12] according to the latest ISUP/WHO 2022 standards, compared the WHO/ISUP grading to the original Fuhrman grading, and applied the latest version of the TNM [25]. Using the data from this re‐evaluation, we updated our previously published prognostic model [12] to evaluate whether ISUP grading, TNM 8th edition, and several novel prognostic factors (LVI, TN, sarcomatoid differentiation, and rhabdoid differentiation) impact the prognostic value of our model.

## Materials and methods

### Study population

For this study, we used the population‐based Netherlands Cohort Study on diet and cancer (NLCS). The NLCS study is a prospective cohort study that has been described in detail elsewhere [26, 27, 28]. In summary, this cohort was initiated in September 1986 and included 120,852 men and women, aged 55–69 years at diagnosis. Follow‐up for cancer occurrence was available through computerized record linkage with the Netherlands Cancer Registry (NCR) and the Automated National Pathological Anatomy Archive (Palga), a national database of pathology reports as described previously [29]. After 20.3 years of follow‐up, 608 histologically confirmed RCC cases were eligible for collection of formalin‐fixed‐paraffin‐embedded (FFPE) tumor tissue from 51 pathology laboratories throughout the Netherlands. Hematoxylin and eosin (H&E) slides of all collected FFPE tumor tissues were originally assessed based on the 2004 WHO classification, and nuclear grading was performed according to the Fuhrman grading system. TNM stage was initially classified according to the 1987 version (3rd edition) [12]. Recently, all tumors were re‐evaluated according to the 2022 ISUP/WHO classification and reassigned according to histological subtype, ISUP grade, TN, sarcomatoid differentiation, rhabdoid features, LVI, TNM version 8th edition, ISUP, and WHO diagnostic criteria [25]. As previously published, 336 ccRCC cases with non‐metastatic disease at diagnosis were available [12]. For 308 (92%) of these, digital scans could be used for re‐evaluation purposes, and these cases were included in the current study. Clinical characteristics of these cases are summarized in Table 1.

**Table 1:** Characteristics of clear cell renal cell cancer patients overall and by promoter methylation of *NEURL*, *NEFH*, *LAD1*, *GREM1*, and *GATA5*

		All ccRCC *N* = 308	*NEURL*	*NEFH*	*LAD1*	*GREM1*	*GATA5*
*N* methylated/*N* total* (%)			95/266 (35.71%)	174/251 (69.32%)	103/278 (37.05%)	120/272 (44.12%)	92/269 (34.20%)
Sex, *N* (%)	Male	180 (58.44)	51 (53.68)	92 (52.87)	64 (62.14)	66 (55.00)	54 (58.70)
	Female	128 (41.56)	44 (46.32)	82 (47.13)	39 (37.86)	54 (45.00)	38 (41.30)
	*p* ^†^		0.396	0.011	0.316	0.255	0.937
Age, mean (SD)		71.12 (6.25)	72.57 (6.43)	71.97 (6.32)	70.70 (6.14)	72.40 (6.15)	72.07 (6.49)
	*p* ^†^		0.033	0.011	0.191	0.009	0.144
Tumor size (mm), mean (SD)		64.6 (31.7)	69.33 (33.17)	64.67 (32.57)	69.45 (33.37)	64.58 (32.30)	73.18 (36.49)
	*p* ^†^		0.097	0.521	0.122	0.945	0.006
ISUP grade, *N* (%)	G1	80 (25.97)	14 (14.74)	40 (22.99)	22 (21.36)	23 (19.17)	16 (17.39)
	G2	132 (42.86)	47 (49.47)	78 (44.83)	36 (34.95)	57 (47.50)	43 (46.74)
	G3	69 (22.40)	22 (23.16)	43 (24.71)	32 (31.07)	30 (25.00)	25 (27.17)
	G4	27 (8.77)	12 (12.63)	13 (7.47)	13 (12.62)	10 (8.33)	8 (8.70)
	*p* ^†^		0.004	0.151	0.011	0.225	0.191
Necrosis, *N* (%)	Present	82 (25.97)	29 (30.52)	52 (29.88)	37 (35.92)	33 (27.5)	28 (30.43)
	Absence	224 (72.72)	65 (68.42)	121 (69.54)	66 (64.08)	86 (71.67)	63 (68.48)
	Not available	2 (0.65)	1 (1.05)	1 (0.57)	–	1 (0.83)	1 (1.09)
	*p* ^†^		0.103	0.142	0.008	0.548	0.214
LVI, *N* (%)	Present	49 (15.91)	15 (15.79)	20 (11.49)	18 (17.48)	16 (13.33)	13 (14.13)
	Absence	257 (83.44)	79 (83.16)	153 (87.93)	85 (82.52)	103 (85.83)	78 (84.78)
	Not available	2 (0.65)	1 (1.05)	1 (0.57)	–	1 (0.83)	1 (1.09)
	*p* ^†^		0.687	0.004	0.781	0.396	0.574
Sarcomatoid, *N* (%)	Present	18 (5.84)	8 (8.42)	9 (5.171)	9 (8.74)	8 (6.67)	5 (5.43)
	Absence	289 (93.83)	87 (91.58)	165 (94.83)	94 (91.26)	112 (93.33)	87 (94.57)
	Not available	1 (0.32)	–	–	–	–	–
	*p* ^†^		0.146	0.656	0.165	0.468	0.942
Rhabdoid, *N* (%)	Present	11 (3.57)	4 (4.26)	5 (2.91)	5 (4.85)	3 (2.5)	4 (4.35)
	Absence	292 (94.80)	90 (95.74)	167 (26.4)	98 (95.15)	115 (95.83)	68 (73.91)
	Not available	5 (1.62)	1 (1.05)	2 (1.14)	–	2 (1.66)	2 (2.17)
	*p* ^†^		0.238	0.917	0.140	0.772	0.192
TNM stage, *N* (%)	I	164 (53.4)	42 (44.21)	95 (54.6)	45 (43.69)	61 (50.83)	38 (41.3)
	II	41 (13.4)	17 (17.89)	23 (13.22)	13 (12.62)	14 (11.66)	20 (21.73)
	III	101 (32.9)	35 (36.84)	55 (31.6)	43 (41.74)	44 (36.66)	33 (35.87)
	IV^‡^	1 (0.32)	1 (1.05)	1 (0.57)	1 (0.97)	–	–
	Not available	–	–	–	1 (0.97)	1 (0.83)	1 (1.09)
	*p* ^†^		0.086	0.473	0.070	0.497	0.007

* Numbers do not add up to 308 due to missing information on methylation status.

^†^ Unmethylated versus methylated.

^‡^ For further analyses TNM stage III and IV were combined.

### DNA isolation, sodium bisulfite conversion, and promoter CpG island methylation analysis

Details on DNA isolation, sodium bisulfite conversion, and methylation analyses for *NEFH*, *NEURL*, *GATA5*, *GREM1*, and *LAD1* are described elsewhere [12]. In brief, genomic DNA from ccRCC tissue was retrieved either by salt‐precipitation or isolated after macro‐dissection using QIAmp DNA Mini Kit (Qiagen, Venlo, the Netherlands) according to the manufacturer's instructions. Sodium bisulfite modification of 500 ng genomic DNA was performed using the EZ DNA Methylation Kit (Zymo Research, Irvine, CA, USA). Promoter CpG island methylation was determined by nested MSP as previously described in detail [12]. To facilitate MSP analysis on DNA derived from FFPE material, DNA was first amplified with flanking PCR primers. All PCR reactions were performed with controls for unmethylated alleles (CpGenomeTM Human Non‐Methylated DNA Standard, Merck Millipore, Burlington, MA, USA or EpiTect control DNA unmethylated, Qiagen), methylated alleles (CpGenomeTM Human Methylated DNA Standard, Merck Millipore or an in‐house prepared *in vitro* methylated DNA control), and a no‐template control without DNA. Ten microliter of each MSP reaction was directly loaded onto 3% agarose gels containing Midori Green Advance DNA Stain (Nippon Genetics Europe, Dueren, Germany) and visualized under UV illumination. Reproducibility of MSP analysis was assessed by repeating 10% of the cases.

### Statistical analysis

Differences in clinicopathological characteristics between subgroups based on TNM stage or methylation status of the five DNA methylation markers were evaluated using *t*‐tests or chi‐square tests where appropriate. Cause‐specific survival (CSS) was defined as the time from cancer diagnosis until RCC‐related death or the end of follow‐up. Analyses were restricted to 10 years after diagnosis as deaths related to RCC are not likely after that period. Univariable survival analyses for TNM staging, ISUP grading, presence of TN, presence of LVI, presence of rhabdoid features, and presence of sarcomatoid features were performed using Kaplan–Meier and log‐rank tests. Due to low numbers in stage IV patients, TNM stage III and IV patients were combined for analysis. Hazard ratios (HR) and corresponding 95% confidence intervals (CI) were determined using Cox proportional hazard models. The previously published prognostic model including five DNA methylation markers (*GREM1*, *GATA5*, *LAD1*, *NEFH*, and *NEURL*), age at diagnosis, sex, Fuhrman grade, tumor size, and TNM stage was compared to a model including the five methylation markers, age at diagnosis, sex, and the new prognostic factors (ISUP grading, TNM 8th edition, presence of TN, presence of LVI, presence of rhabdoid differentiation, presence of sarcomatoid differentiation). This was done to evaluate whether these prognostic factors would improve the model. The preferred model was the one with the lowest AIC and the highest c‐statistic (c‐statistic was leading if the highest c‐statistic did not have the lowest AIC) [30]. All analyses were performed using the statistical software STATA 17.0.

## Results

Table 1 shows the baseline characteristics of the patients included in this study. Most patients in the total population were male (180/308, 58.44%), the mean age at diagnosis was 71.12 ± 6.25 years, and the mean tumor size was 64.6 ± 31.7 mm. ISUP grading could be established for all 308 cases; most cases were G2 (*n* = 132, 42.86%). Necrosis was present in 82 patients (25.97%), LVI in 49 patients (15.9%), sarcomatoid features in 18 patients (5.84%), and rhabdoid features in 11 patients (3.6%). For all 308 patients, we were able to re‐evaluate the TNM stage according to the 8th edition; most cases were stage I (*n* = 164, 53.4%) or stage III (*n* = 101, 32.9%). The percentages of methylation for the five DNA methylation markers were as previously published [12]; *NEURL* (35%), *NEFH* (69%), *LAD1* (37%), *GREM1* (44%), and *GATA5* (34%) (Table 1). Previously, we described a statistically significant association between methylated *LAD1* and *NEURL*, and Fuhrman grade (*p* = 0.004 and 0.001, respectively), and a borderline statistically significant association between methylated *LAD1* and TNM Stage 3rd edition (*p* = 0.058) [12]. In part, this was also seen using ISUP grading and TNM stage 8th edition, showing a statistically significant association between methylated *LAD1* and *NEURL*, and ISUP grading (*p* = 0.011 and 0.004, respectively) and a borderline significant association between methylated *LAD1* and TNM stage 8th edition (*p* = 0.070). However, in this study, we also observed a borderline statistically significant association between methylated *NEURL* and *GATA5*, and TNM stage 8th edition (*p* = 0.086 and 0.007, respectively) that was not seen in our previous study. Novel pathological prognostic factors (necrosis, LVI, and the presence of sarcomatoid and/or rhabdoid differentiation) were strongly associated with TNM stage as expected and were more often present in the higher TNM stages (*p*_necrosis_ 0.007, *p*_LVI_ 0.008, *p*_sarcomatoid_ 0.001, *p*_rhabdoid_ 0.002) (Table 2).

**Table 2:** Association of the TNM 8th edition with the new ISUP prognostic features

	TNM stage		
	I	II	III and IV
Necrosis, *N* (%)			
Present	32 (39.02)	15 (18.29)	35 (42.70)
Absent	132 (59.19)	25 (11.21)	66 (29.60)
*p*			0.007
LVI, *N* (%)			
Present	17 (34.69)	7 (14.29)	25 (51.02)
Absent	147 (57.42)	33 (12.89)	76 (29.70)
*p*			0.008
Sarcomatoid, *N* (%)			
Present	3 (16.67)	2 (11.11)	13 (72.22)
Absent	161 (55.90)	38 (13.19)	89 (30.90)
*p*			0.001
Rhabdoid, *N* (%)			
Present	1 (9.09)	1 (9.09)	9 (81.82)
Absent	161 (55.33)	40 (13.75)	90 (30.90)
*p*			0.002

**Figure 1:**
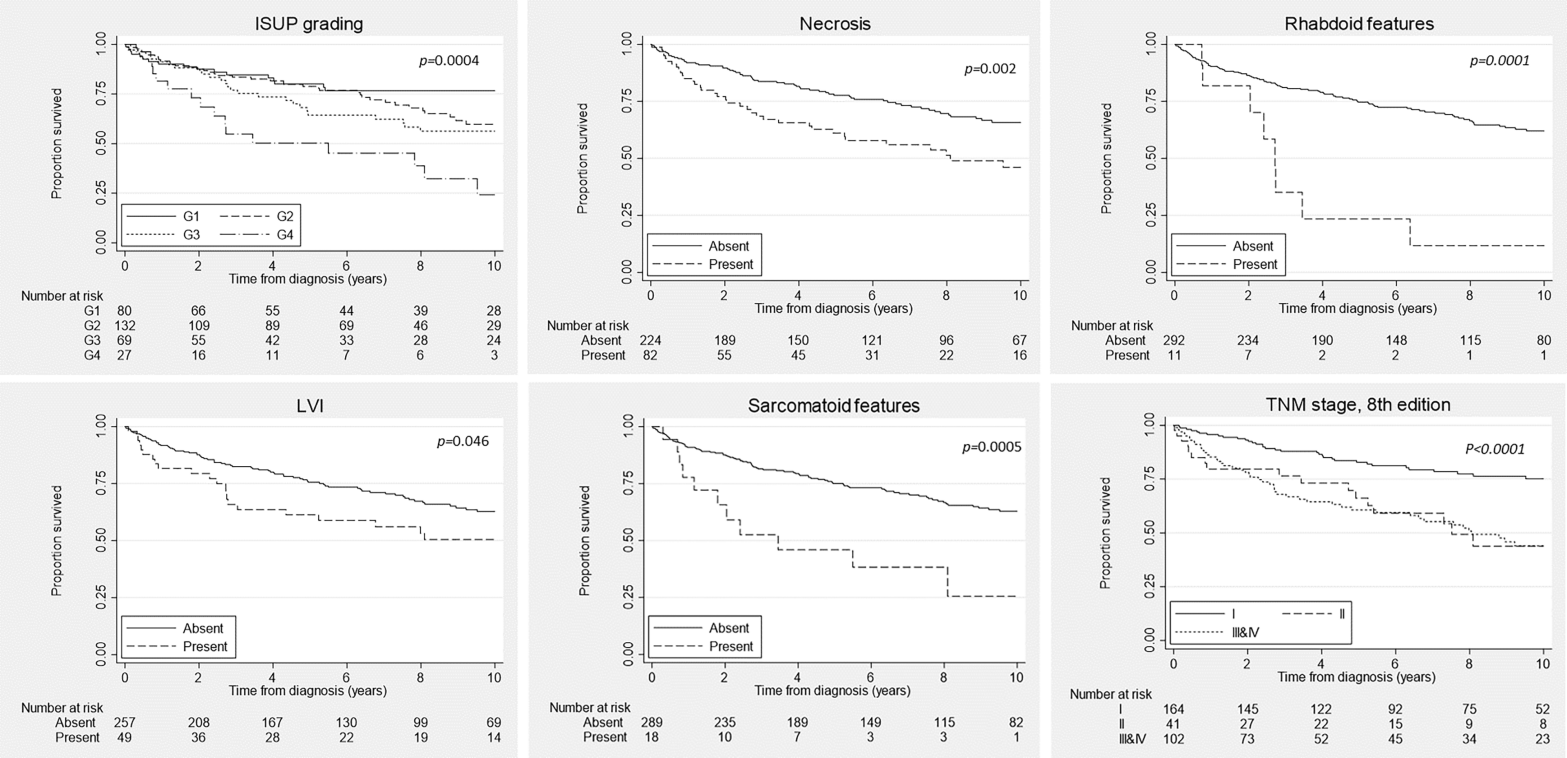
Overall cause‐specific survival curves for ISUP grading, necrosis, rhabdoid differentiation, LVI, sarcomatoid differentiation, and the TNM 8th edition in the population‐based ccRCC series. The *p* value is estimated by log‐rank test.

To evaluate the prognostic effect of ISUP grading, necrosis, LVI, sarcomatoid differentiation, rhabdoid differentiation, and the TNM 8th edition, Kaplan–Meier curves were created (Figure 1). All prognostic factors described in the new ISUP standards had prognostic value. As expected, a higher ISUP grade (grade 3 and 4) was associated with a poorer prognosis (*p*_ISUP_ = 0.0004). Similarly, the presence of necrosis, LVI, and sarcomatoid and/or rhabdoid differentiation was associated with a poor prognosis (*p*_necrosis_ = 0.002, *p*_LVI_ = 0.046, *p*_sarcomatoid_ = 0.0005, *p*_rhabdoid_ = 0.0001). In line with expectations, TNM stage (8th edition) was a very strong prognostic factor (*p*_TNM_8th_edition_ < 0.0001). Survival analyses adjusted for age at diagnosis, sex, and TNM stage also indicated the prognostic value of ISUP grading, although not statistically significant for all grades: HR_G2_ 1.23 (95% CI 0.68–2.21), HR_G3_ 1.49 (95% CI 0.79–2.81), and HR_G4_ 2.58 (1.25–5.33) as compared to ISUP G1. Age, sex, and TNM stage‐adjusted HRs for the presence of necrosis, LVI, and sarcomatoid or rhabdoid differentiation showed the same trend: HR_necrosis_ 1.68 (95% CI 1.11–2.54), HR_LVI_ 1.27 (95% CI 0.77–2.09), HR_sarcomatoid_ 2.02 (95% CI 1.06–3.85), and HR_rhabdoid_ 3.00 (95% CI 1.40–6.45).

Previously, we developed a model containing five DNA methylation markers, age at diagnosis, sex, TNM stage (3rd edition), Fuhrman grade, and tumor size that showed slightly better prognostic value compared to a clinical model containing solely clinicopathological characteristics (age at diagnosis, sex, TNM stage (3rd edition), Fuhrman grade and tumor size) [12]. Considering that the previous TNM version and grading system were used in this previous clinical model, we first wanted to assess the prognostic value of the clinical model according to the new TNM stage (8th edition) and the ISUP grading system. The clinical model using the novel grading and staging systems performed slightly better (AIC 592, c‐statistic 0.63) compared to the clinical model using the previous grading and staging systems (AIC 595, c‐statistic 0.63). Adding necrosis or LVI as separate prognostic factors or combined did not further improve the original clinical model (AIC 594, c‐statistic 0.64; AIC 596, c‐statistic 0.62; AIC 595, c‐statistic 0.65, respectively). Along this line, adding sarcomatoid or rhabdoid differentiation, separately or combined, did not influence model performance (AIC 593, c‐statistic 0.63; AIC 593, c‐statistic 0.63; AIC 591, c‐statistic 0.65, respectively). Adding the five DNA methylation markers to the clinical model with the novel staging and grading systems slightly improved the model (AIC 584, c‐statistic 0.70). This slight improvement was comparable to the model using the older grading and staging systems (AIC 586, c‐statistic 0.70). The addition of necrosis and LVI did not further improve these results (AIC 586, c‐statistic 0.71 and AIC 587, c‐statistic 0.71, respectively) (Table 3).

**Table 3:** Comparison of model performance and fit

	Clinical model 1*	Clinical model 2^†^
Basic (A)		
*n*	198	198
df	9	8
AIC	595	592
c‐statistic	0.62	0.63
(A) + necrosis and LVI (B)		
*n*	198	198
df	10	10
AIC	594	593
c‐statistic	0.64	0.65
(A) and DNA methylation markers (C)		
*n*	198	198
df	13	13
AIC	586	584
c‐statistic	0.70	0.70
(B) and DNA methylation markers (D)		
*n*	198	198
df	15	15
AIC	587	586
c‐statistic	0.71	0.71

Numbers in the table refer to the number of cases included in the analysis (*n*), degrees of freedom (df), Akaike information criterion (AIC), and the Harrel's c‐statistic (c‐statistic). Lower number of patients included in the analysis due to missing data on methylation status of the included genes.

* Model containing age at diagnosis, sex, TNM stage (1987), Fuhrman grade, and tumor size.

^†^ Model containing age at diagnosis, sex, TNM stage (8th edition), ISUP grading, and tumor size.

## Discussion

Previously, we developed a prognostic biomarker model containing five DNA methylation markers (*GREM1*, *GATA5*, *LAD1*, *NEFH*, and *NEURL*) in combination with clinicopathological characteristics including age at diagnosis, sex, Fuhrman grade, tumor size, and TNM 3rd edition staging system. This prognostic model showed a slightly better prognostic value in two different patient series as compared to a model with solely clinicopathological risk factors [12]. However, the understanding of the morphology, molecular, and clinical features of RCC tumors has developed [20, 31, 32] leading to new RCC staging and grading systems and to the identification of novel prognostic features. Our data show that these novel features, such as necrosis, LVI, and the presence of sarcomatoid and rhabdoid differentiation, are more often present in higher TNM stages and are associated with a poorer prognosis in ccRCC. However, a prognostic model containing ISUP grading and TNM 8th edition, together with age at diagnosis, sex, and tumor size, performed comparably to a prognostic model using the Fuhrman grading system and TNM 3rd edition [33]. Adding necrosis and LVI as prognostic factors did not further improve this prognostic model, indicating that, although these individual factors might influence prognosis, they do not add additional prognostic information to a model containing several other established factors. The prognostic model containing the five DNA methylation markers showed comparable results when adding ISUP grading and the TNM 8th as compared to the model with Fuhrman grading and the TNM 3rd edition and seemed to perform slightly better than the model with clinicopathological characteristics alone. The addition of necrosis and LVI to the model, however, did not further improve this prognostic performance.

Despite the recent development of different prognostic models and/or tools, such as the SSIGN and the UISS, risk stratification of RCC patients is still suboptimal, especially due to tumor heterogeneity, if only small biopsy samples were used to gain information on tumor characteristics. Current models thus leave room for improvement, and better identification of high‐risk tumors is still necessary [34]. With this in mind, several novel prognostic features have been proposed over recent years, including necrosis, LVI, and the presence of sarcomatoid or rhabdoid differentiation [35, 36, 37]. Although the prognostic value of these features on their own seems established, they did not show any additional prognostic value when added to the multivariate models in our study. This is consistent with previously reported results suggesting that in grade 4 RCC tumors, the presence of rhabdoid differentiation was not associated with survival. In contrast, in the same study, sarcomatoid differentiation has been shown to be significantly associated with survival [38].

Despite the fact that ISUP grading and the TNM 8th edition were proposed for application in the clinic as they would provide more accurate prognostic information compared to their previous versions [7, 19, 20, 39], we have seen comparable prognostic performances between our previously published prognostic model including Fuhrman grading and the TNM 3rd edition and the updated clinical model including the ISUP/WHO standards and TNM 8th edition. In contrast to Fuhrman grading, the ISUP grading system captures different aspects such as nucleolus identification to determine WHO/ISUP grade 1–3 and the presence of polymorphic giant tumor cells, sarcomatoid or rhabdoid differentiation features to assign grade 4. Consequently, all cases that showed sarcomatoid or rhabdoid differentiation were graded as ISUP grade 4 while these cases did not always have a high grade according to the Fuhrman grading. This could lead to a different classification of individual patients, as also described previously [25]. This might clarify why incorporating the new prognostic features did not enhance the performance of the prognostic model after adjusting for the tumor grade. Nevertheless, for the model performance, Fuhrman grading and ISUP grading seemed to perform similarly in our study.

The five DNA methylation markers, on the other hand, do seem to slightly improve the model, indicating that molecular biomarkers could be a valuable contribution to improve risk stratification in RCC. These findings can offer valuable insights, particularly in cases where biopsies may not fully represent the complexity of the tumor. Although renal biopsies are thought to be reliable diagnostic tools [40], these biopsies are often taken from a small portion of the tissue and hence can be limited in providing sufficient and comprehensive (pathological) information within a tumor. DNA methylation information from these biopsies may be more informative, although even for these marker types, single tumor‐biopsy samples may underestimate DNA methylation rates due to tumor heterogeneity [41]. For the five DNA methylation markers, it would have been interesting to see if different tissue blocks would yield different DNA methylation results. Unfortunately, as multiple tissue blocks per patient were not available, we were not able to test for tumor heterogeneity. Nevertheless, DNA methylation markers could also be useful tools in the management of small renal masses (SRMs; kidney tumors <4 cm). Distinguishing between benign and malignant SRMs can be challenging based on imaging alone, and DNA methylation markers can potentially help in assessing the aggressiveness of the SRMs and stratify SRMs into categories [42].

In our study, there appeared to be no differences in prognostic performance between the models incorporating the new or the old versions of the grading and staging systems. In part, this could be explained by the resemblance between the previous and current grading and staging systems. For example, the most reliable and reproducible criterion of the Fuhrman grading is the nucleolar size, and the same applies for the ISUP grading system. Moreover, the integration of rhabdoid or sarcomatoid differentiation was also previously practiced in clinic, though only officially approved after the ISUP consensus. Nevertheless, the re‐evaluation of previously published models is valuable to ensure the applicability and clinical relevance of these models. In addition, it will allow for a better comparison between individual studies that can help in the development of prognostic tools and ensure that they align with the latest understanding of RCC tumor behavior and characteristics.

This study has some limitations that could have influenced the results. The patients in the included population‐based cohort were initially included in 1986 and were thus diagnosed and treated using the common practices at that time. We performed our re‐evaluation with all available information that was gathered at that time. However, due to changed clinical practices, it is possible that specific information was not registered (or measured) at that time or is currently detected more accurately. Because of this, we might have missed crucial information in specific cases that could partly explain why adding prognostic information had limited influence on model performance. Furthermore, for the evaluation of the novel prognostic factors, we used (digital) slides that were originally chosen by the pathologists as the ones being most representative of the tumor. These slides might not necessarily be the most representative slides for the specific prognostic factor that was evaluated and we could have missed essential information because of this.

In summary, our prognostic model containing the DNA methylation biomarkers remained the best performing model, indicating that adding DNA methylation biomarkers could help identify high risk RCC patients in clinical practice. Updating this model using the novel staging and grading systems did not alter the prognostic performance. Similarly, despite the individual prognostic value of features such as necrosis and LVI, adding these factors did not improve the prognostic performance of the model. Further validation of our prognostic biomarker model in large, preferably prospective, patient series is however warranted to establish its clinical value.

## Author contributions statement

IS, MvE, LJS and KMS conceived and designed the study. SO and IS collected data. IS, AM, MMLLB, CAHVDK and AZH performed the pathology review and interpretation of the pathological data. SO, IS and KMS analyzed and interpreted data and wrote the manuscript. MvE and KMS obtained funding. IS, LJS and KMS supervised the study. All authors corrected and agreed to the final version of the manuscript.

## Data Availability

The datasets generated and/or analyzed during the current study are not publicly available because the informed consent does not allow for that.
